# XRCC1 Coordinates Disparate Responses and Multiprotein Repair Complexes Depending on the Nature and Context of the DNA Damage

**DOI:** 10.1002/em.20663

**Published:** 2011-07-22

**Authors:** Audun Hanssen-Bauer, Karin Solvang-Garten, Ottar Sundheim, Javier Peña-Diaz, Sonja Andersen, Geir Slupphaug, Hans E Krokan, David M Wilson, Mansour Akbari, Marit Otterlei

**Affiliations:** 1 Department of Cancer Research and Molecular Medicine, Faculty of Medicine, Norwegian University of Science and TechnologyTrondheim, Norway; 2 Laboratory of Molecular Gerontology, National Institute on AgingNIH, Baltimore, Maryland, USA

**Keywords:** base excision repair, micro-irradiation, PARP inhibitors, PCNA, DNA repair complexes

## Abstract

XRCC1 is a scaffold protein capable of interacting with several DNA repair proteins. Here we provide evidence for the presence of XRCC1 in different complexes of sizes from 200 to 1500 kDa, and we show that immunoprecipitates using XRCC1 as bait are capable of complete repair of AP sites via both short patch (SP) and long patch (LP) base excision repair (BER). We show that POLβ and PNK colocalize with XRCC1 in replication foci and that POLβ and PNK, but not PCNA, colocalize with constitutively present XRCC1-foci as well as damage-induced foci when low doses of a DNA-damaging agent are applied. We demonstrate that the laser dose used for introducing DNA damage determines the repertoire of DNA repair proteins recruited. Furthermore, we demonstrate that recruitment of POLβ and PNK to regions irradiated with low laser dose requires XRCC1 and that inhibition of PARylation by PARP-inhibitors only slightly reduces the recruitment of XRCC1, PNK, or POLβ to sites of DNA damage. Recruitment of PCNA and FEN-1 requires higher doses of irradiation and is enhanced by XRCC1, as well as by accumulation of PARP-1 at the site of DNA damage. These data improve our understanding of recruitment of BER proteins to sites of DNA damage and provide evidence for a role of XRCC1 in the organization of BER into multiprotein complexes of different sizes. Environ. Mol. Mutagen. 2011. © 2011 Wiley-Liss, Inc.

## INTRODUCTION

X-ray repair cross-complementing protein 1 (XRCC1) has no known enzymatic activity but is involved in single-strand break repair (SSBR) and base excision repair (BER), both considered to be part of the same pathway. Base damage of different types, abasic sites (AP sites), and SSBs are continuously formed in the human genome in numbers exceeding 10^4^ per day. Repair of such lesions involves a number of proteins in addition to XRCC1 and is mainly carried out by the BER/SSBR pathway. BER takes place via short patch (SP, single nucleotide insertion) or long patch (LP, usually insertion of 2–8 nucleotides) repair [Frosina et al.,^1999^].

Xrcc1^−/−^ mice are embryonic lethal [Tebbs et al.,^1999^] and Xrcc1 deficient rodent cell lines display reduced SSBR capacity, increased frequency of sister chromatid exchange, and sensitivity to several types of DNA damaging agents, particularly those that generate SSBs and base lesions [Caldecott,^2001^]. Inefficient XRCC1-associated SSBR contributes to neurodegenerative disease in humans [El-Khamisy et al.,^2005^; Hirano et al.,^2007^]. Xrcc1^−/−^ cells were recently shown to have a slightly reduced rate of repair of uracil in DNA, but not repair of AP sites [Akbari et al.,^2010^].

A number of protein–protein interactions and posttranslational modifications of proteins involved in BER have been reported [Fan and Wilson,^2005^; Prasad et al.^2007^; Hagen et al.,^2008^]. XRCC1 likely modulates DNA repair by its ability to interact with several BER proteins including DNA glycosylases [Marsin et al.,^2003^; Campalans et al.,^2005^; Akbari et al.,^2010^], AP endonuclease-1 (APE-1) [Vidal et al.,^2001^], DNA polymerase β (POLβ) [Caldecott et al.,^1996^], DNA ligase III (Lig III) [Caldecott et al.,^1994^], proliferating cell nuclear antigen (PCNA) [Fan et al.,^2004^], poly (ADP-ribose) polymerase 1 (PARP-1) [Masson et al.,^1998^], PARP-2 [Schreiber et al.,^2002^], and polynucleotide kinase (PNK) [Whitehouse et al.,^2001^]. However, whether these interactions can result in the formation of large and stable complexes remains elusive.

*In vitro* as well as *in vivo* results from live cell fluorescence energy transfer (FRET) analysis suggested that XRCC1 forms multimers [Fan et al.^2004^; Beernink et al.^2005^; Akbari et al.^2010^]. Different XRCC1 associated complexes for repair of SSBs have also been identified by immunoprecipitation experiments [Luo et al.,^2004^; El-Khamisy et al.,^2005^]. Recently, XRCC1 was identified in replication associated complexes and analysis of the content and function of these complexes showed that they contain many of the above-mentioned BER proteins, as well as some replication proteins and could efficiently perform repair of AP sites and uracil [Parlanti et al.,^2007^; Akbari et al.,^2010^]. However, less is known about stable multiprotein XRCC1 complexes in cells outside S-phase, even though various numbers of XRCC1-foci in these cells suggest the presence of preformed XRCC1 complexes [Fan et al.,^2004^; Akbari et al.,^2010^]. In a previous report, fractionation of whole cell extracts by gel filtration identified only XRCC1-Lig III heterodimers of approximately 100–200 kDa in size (corresponding to the sum of the relative molecular weight of Lig III and XRCC1 monomers and heterodimers) [Parsons et al.,2005a]. Notably, in this and several other studies that attempted to isolate XRCC1 complexes, cell extracts did not include the chromatin-bound fraction of proteins.

Our aim for this study was to explore the role of XRCC1 in the organisation of BER-proficient complexes in freely cycling cells, in the absence or presence of exogenously induced DNA damage. We present a model of three modes of BER involving XRCC1.

## MATERIALS AND METHODS

### Chemicals and Antibodies

The synthetic oligonucleotides were from Eurogentech (Belgium), [α-^33^P]dCTP and [α-^33^P]dTTP (3000 Ci/mmol) from Amersham Biosciences, restriction enzymes from New England BioLabs, paramagnetic protein-A beads from Dynal (Norway), MMS and H_2_O_2_ from Sigma-Aldrich. α-POLβ IgG (ab3181), α-DNA Lig III IgG (ab587), α-XRCC1 IgG (ab1838), α-γH2AX antibody (ab2893) and α-GFP IgG (ab290) were from Abcam (UK). Other antibodies used were α-PNK IgG (MAB-005, Cytostore, USA), α-PCNA IgG (PC10, Santa Cruz, USA), in-house affinity purified rabbit polyclonal antibodies (IgG fraction) raised against GFP protein, and UNG2 [Slupphaug et al.,^1995^]. Secondary antibodies (polyclonal rabbit α-mouse IgG/HRP or peroxidase-labelled polyclonal swine α-rabbit IgG) were from Dako Cytomation (Denmark). IgGs were crosslinked to protein-A magnetic beads according to the procedure provided by New England Biolabs. The neutralising α-POLβ antibody was a generous gift from Dr. Samuel H. Wilson (Laboratory of Structural Biology, NIH). 4-amino-1, 8-naphthalimide (4-AN) (Sigma) and *N*- (6-oxo-5,6-dihydrophenanthridin-2-yl)−2-(*N*,*N*-dimethylamino)acetamid (PJ34) (Santa Cruz) were dissolved to 10 m*M* in DMSO.

### Cloning of Fusion Constructs

pEC/YFP-PCNA, pHcRed-PCNA (small protein from the reef coral *Heteractis crispa*), pXRCC1-EYFP, and pEYFP-FEN-1 have been described [Aas et al.^2003^; Fan et al.,^2004^; Opresko et al.,^2004^; Sharma et al.,^2004^]. The NdeI- (blunted)-SalI fragment of POLβ from pT7-7 [Nguyen et al.,^2000^] was ligated into XhoI (blunted)-SalI digested pECFP and pEYFP-C1 plasmids (Clontech). The PNK open reading frame (encoding 563 amino acids) was PCR amplified and subcloned into the NcoI and HindIII sites of the pBlueBacHis2 B vector (InVitrogen). The PNK-containing BglII-HindIII fragment was transferred into the pEC/EYFP-C1 plasmid to prepare an N-terminal fusion product. All constructs were verified by sequencing. pEYFP-PARP-1 was prepared by subcloning from the pDsRed1C1-PARP-1 construct [von Kobbe et al., ], kindly provided by Dr. Cayatano von Kobbe, NIH, NIA, using the Sal I site.

### Cell Cultures

Cells were transfected with different fusion constructs using calcium phosphate (Profection Promega) or Fugene6 (Roche). HeLa and CHO (CHO AA8, CHO EM9) cells stably expressing XRCC1-EYFP or EYFP were prepared by transfection followed by cell sorting or cloning by dilution and prolonged culturing in selective DMEM medium (Gibco) containing 10% fetal calf serum, gentamycin (0.1 mg/ml, Gibco), glutamine (1 mM), fungizone (2.5 μg/l), and geneticin (G418, 0.4 mg/ml, added two days after tranfection). For transient transfections, cells were examined after 16–24 hr. Untransfected cells were cultured in the same medium without geneticin. Cells were treated with 10 μ*M* 4-AN or PJ34 60 min at 37°C prior to micro-irradiation experiments.

### Confocal Microscopy

Fluorescent images of living cells cotransfected with ECFP, EYFP, and HcRed constructs (1 μm optical slide thickness) were produced using a Zeiss LSM 510 Meta laser scanning microscope equipped with a Plan-Apochromate 63× 1.4 oil immersion objective. Three colour images were taken using three consecutive scans with the following settings: ECFP-excitation at λ = 458 nm, detection at λ = 470–500 nm, EYFP-excitation at λ = 488 nm, at λ = 530–600 nm and HcRed-excitation at λ = 543 nm, detection at λ > 615 nm. Alexa fluor 647 was excited with 633-nm laser and emission detected at > 650 nm.

### γH2AX Staining

Cells were micro-irradiated, fixed with 2% paraformaldehyde for 10 min on ice, washed once with PBS, permeabilized with methanol at −20°C for 20 min, washed once with PBS-FCS (2% fetal calf serum in PBS) and blocked by incubation in PBS-FCS for 30 min prior to incubation with primary rabbit γH2AX antibody diluted in PBS-FCS (1:200) and incubated at 4°C overnight. After washing the cells, they were incubated with Alexa fluor 647 goat anti-rabbit (1:2000).

### 405-nm Micro-Irradiation

A Zeiss 405-nm diode laser was focused through a 63× 1.4 Plan-Apochromate Oil DIC objective to a diffraction-limited spot size in a LSM 510 Meta microscope. The 405-nm diode output was measured to 30 mW using a FieldMaster GS energy meter (Coherent Inc.) with a low power probe. We used low dose, which recruited POLβ and PNK (10 (HeLa) or 60 (CHO) laser beam iterations; or high dose (150 (HeLa) or 600 (CHO) laser beam iterations), which also recruited PCNA and FEN-1. Time of speed was 1.27 μsec/pixel over a 50 × 2 pixel area in the cell nucleus. Time lapse image acquisition started one scan prior to the micro-irradiation. Signal intensities were measured using the LSM 510 Meta operating software version 4.2. The relative signal strength of the foci were obtained by dividing average foci signal strength with average signal strength measured in a nonirradiated, equally sized region of the nucleus. Only cells with similar signal intensities were analyzed. Size bars on the image equals 5 μm.

### Whole Cell Extracts

Harvested cells were washed once in PBS, suspended in 8× packed cell volume in buffer I (20 m*M* HEPES pH 7.9, 1.5 m*M* MgCl_2,_ 100 m*M* KCl, 0.2 m*M* EDTA, 20% (v/v) glycerol, 0.5% Nonidet P-40, 1 m*M* DTT, and 1 × Complete protease inhibitor, Roche) containing 5 μl Omnicleave Endonuclease (200 U/μl Epicentre Technologies, WI) and sonicated. DNase/RNase cocktail I (2 μl of Omnicleave Endonuclease, 1 μl Benzonase (250 U/ml, Novagene, Ge), 10 μl RNase (10 mg/ml, Sigma-Aldrich), 1 μl DNase (10 U/μl, Roche), and 1 μl Micrococcal Nuclease (100-300 U/mg, Sigma-Aldrich) per 30 mg cell extract) was added to the homogenate and incubated for 30 min at room temperature (RT) before incubation at 4°C overnight during dialysis against buffer II (20 m*M* HEPES pH 7.9, 1.5 m*M* MgCl_2,_ 100 m*M* KCl, 0.2 m*M* EDTA, 10% (v/v) glycerol, 1 m*M* DTT, and 0.1 × Complete protease inhibitor, Roche). The extract was cleared by centrifugation at 14,000 × *g* prior to IP with anti-EYFP coupled beads.

### Gel Filtration

Cell extracts for gel filtration were prepared from isolated cell nuclei. Cells were suspended in lysis buffer (10 m*M* HEPES pH 7.9, 100 m*M* KCl, and 1.5 m*M* MgCl_2_) and incubated for 15 min on ice. The cells were disrupted with a Dounce homogenizer (25–30 tight pestle strokes) and the nuclei were collected (at 650 × *g* for 10 min at 4°C). Nuclei were suspended in 2× packed nuclei volume of buffer I and sonicated. DNase/RNase cocktail I was added, followed by incubation for 1 hr at RT and overnight at 4°C. The extract was centrifuged at 14,000 × *g* and the supernatant was filtered (0.2 μm) prior to loading onto the gel filtration column; 1.5–3 ml of filtered cell extract was loaded on a Sephacryl S-300 HR column using an Äkta fast protein liquid chromatography (FPLC) system (GE Healthcare) at 4°C. The DNA fragments in the extracts after sonication, DNase treatment, and filtration were smaller than 100 base pairs as determined by agarose gel and ethidium bromide staining. The column was equilibrated and run at a flow rate of 0.5 ml/min using buffer II. Fractions of 5 ml were collected and used for IP. High molecular weight and low molecular weight gel filtration calibration kits from GE Healthcare were used as molecular weight (MW) indicators.

### Immunoprecipitation (IP) and Western Blot Analysis

For IP, antibodies that were covalently linked to protein-A paramagnetic beads were incubated with the respective extracts in 5 ml of IP solution at 4°C overnight under constant rotation. The beads were collected and suspended in 1 ml of 10 m*M* Tris—HCl pH 7.5, 100 m*M* KCl (IP of total cell extracts) or buffer II (gel filtration) and washed 4× in 1 ml of these buffers. The beads were used in the BER assay (described below) or suspended in loading buffer, heated, separated on Bis-Tris—HCl NuPAGE ready gels (4–12%, Invitrogen) and transferred to PVDF membranes (Immobilon, Millipore). The membranes were blocked in 5% low fat dry milk in PBST (PBS with 0.1% Tween 20), incubated with primary antibodies in 1% dry milk at 4°C overnight, and incubated 1 hr with secondary antibodies. Membranes were then treated with Chemiluminescence reagent (SuperSignal West Femto Maximum, PIERCE) and the proteins visualised using a Kodak Image Station 2000R.

### BER Assay

We prepared DNA substrates for the BER assay containing uracil or a synthetic analogue of an AP site (3-hydroxy-2-hydroxymethyltetrahydrofuran, THF) at a single position (Supporting Information Figure S1C). A normal AP site (indicated as X in Supporting Information Figure S1C) was generated by incubating uracil-containing DNA substrate with purified catalytic domain of UNG [Slupphaug et al.,^1995^]. BER assays were carried out essentially as described [Frosina et al.,^1999^; Akbari et al.,^2004^] with some modifications. Specifically, we used 10 μ*M* dATP, 10 μ*M* dGTP and 10 μ*M* dTTP, 62.5 n*M* dCTP and 8.3 n*M* [α-^33^P]dCTP (5 μCi/pmol dCTP). The incubation time was 15 min at 32°C. The repaired DNA substrate was digested with the indicated restriction enzymes and analysed by denaturing polyacrylamide gel electrophoresis followed by phosphor imaging (Fuji, BAS-1800II) of the dried gel. We used AP:A DNA substrate (containing an AP site at position 5′ adjacent to X), [α-^33^P]dTTP and XbaI/HincII digestion for analysis of first nucleotide incorporation, or [α-^33^P]dCTP and XbaI/HincII digestion for identification of second nucleotide. For comparison of normal AP site (AP:A) and THF, we used [α-^33^P]dTTP and BamHI/Pst I digestion to detect first nucleotide insertion and amount of ligated product. Use of the same substrates with [α-^33^P]dCTP and BamHI/PstI digestion detected incorporation of second nucleotide and amount of ligated product.

## RESULTS

### Gel Filtration of Nuclear Extracts Identifies XRCC1-EYFP in Complexes with Large Differences in Size and Composition

XRCC1 is associated with the replication machinery where it directly interacts with PCNA [Fan et al.,^2004^] and UNG2 [Akbari et al.,^2010^]. However, XRCC1-foci are also detectable in nontreated non-S-phase cells, and these foci contain neither PCNA nor UNG2 [Fan et al.,^2004^; Akbari et al.,^2010^]. The content and nature of XRCC1 complexes in such foci have not yet been described. Here, we explore the presence, size, composition, and recruitment of XRCC1 and its interacting proteins in different complexes in freely cycling, nontreated cells.

**Fig. 1:**
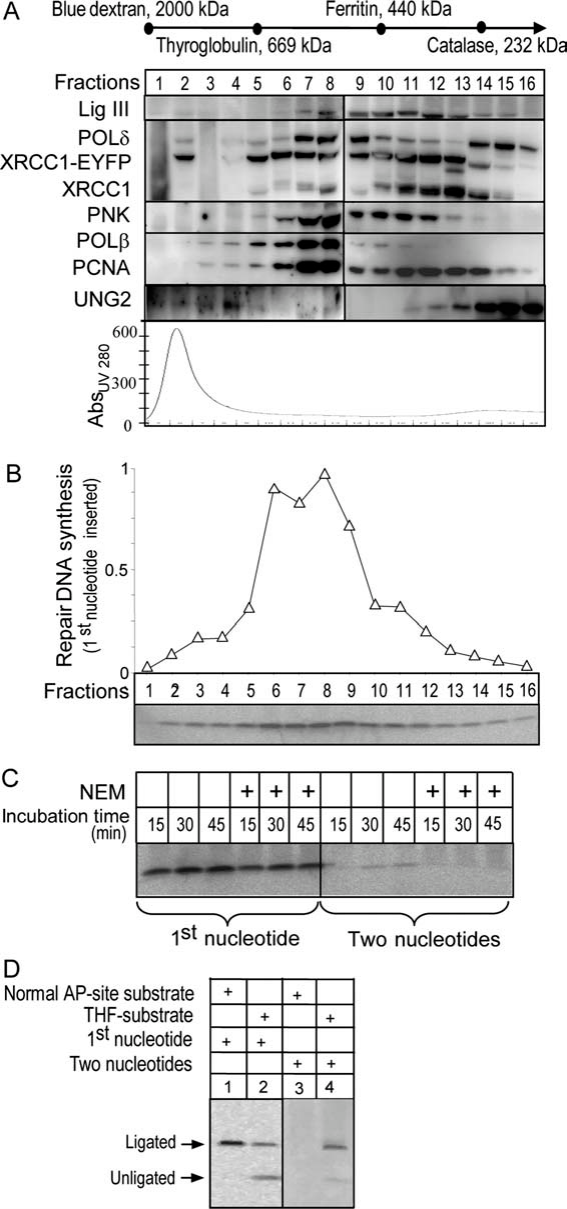
Western blot analysis of immunoprecipitates from size fractionated XRCC1-EYFP expressing HeLa nuclear extract and functional BER analyses of XRCC1-EYFP complexes. (**A**) The gel filtration standards (blue dextran, thyroglobin, ferritin, and catalase) indicate the molecular weight of the fractions. The total protein content in each fraction is illustrated as a curve of ultraviolet light (UV) absorption (280 nm). Immunoprecipitates from fractions 1 to 16 were prepared using α-GFP couplet magnetic beads and subjected to Western blot. (**B**) DNA repair synthesis analysis of the immunoprecipitates from gel filtration fractions 1–16 using AP:G substrate (illustrated in Supporting Information Figure S1C) and [α-^33^P]dCTP, followed by digestion of purified DNA with XbaI/HincII to detect incorporation of the first nucleotide. The *y* axis shows repair synthesis as arbitrary units. Shown below the fraction numbers is a representative gel depicting the labeled DNA product. (**C**) Time-dependent inhibition of DNA repair synthesis activity of XRCC1-EYFP immunoprecipitates from un-fractionated XRCC1-EYFP expressing HeLa nuclear extract by NEM (10 mM). We used AP:A DNA substrate (containing an AP site at position 5′ adjacent to X shown in Supporting Information Figure S1C), [α-^33^P]dTTP and digestion of the product with XbaI/HincII to measure first nucleotide incorporation, or [α-^33^P]dCTP and digestion of DNA with XbaI/HincII to detect incorporation of second nucleotide. (**D**) BER was carried out for 15 min using a normal AP site (AP:A) or THF (THF:A) DNA substrates. Using [α-^33^P]dTTP in the assay and digestion of the product with BamHI/PstI detects the first nucleotide incorporation and amount of ligated product. Use of the same substrates and [α-^33^P]dCTP and digestion of the DNA with the same enzymes detects incorporation of the second nucleotide. One representative set of data from three experiments is shown.

We first established a HeLa cell line stably expressing near endogenous levels of EYFP-tagged functional XRCC1 (XRCC1-EYFP) (Supporting Information Figures S1A and S1B). From these cells, we prepared DNase/RNase digested total nuclear extracts to explore whether we could identify XRCC1 in large stable complexes. We applied gel filtration to separate proteins and protein complexes according to their relative molecular weight and collected fractions containing proteins in the range of ∼70–2000 kDa. To be able to detect XRCC1 associated proteins in the different fractions, we had to enrich the fractions by immunoprecipitation. XRCC1-EYFP and its associated proteins were pulled down from each fraction using α-GFP coupled beads and analyzed for protein content and ability to repair AP sites (Figs. 1A and 1B). Importantly, these studies revealed the presence of XRCC1-EYFP in complexes of different sizes and composition. We found that immunoprecipitates from fractions 5 through 11 (approximately 700–350 kDa) had the highest capacity to cleave at an AP site and incorporate nucleotides (Figs. 1A and 1B). These immunoprecipitates contained detectable Lig III, POLδ, XRCC1, PNK, POLβ, and PCNA. We could not detect APE-1 by Western analysis (data not shown); however, the immunoprecipitates clearly contained AP-endonuclease activity suggesting the presence of the protein (Fig. 1B).

UNG2 and XRCC1 were found in both large and small complexes (Fig. 1A, fractions 2–4 and 12–16). We have shown that XRCC1 interacts directly with UNG2, that UNG2 and XRCC1 are in the same complex only in S-phase, and that this complex is large and contains many proteins [Akbari et al.,^2010^]. Thus, the small UNG2-XRCC1 complexes may be an example of protein interactions and complexes not present *in situ*, but an artefact from the IP procedure as seen previously [Akbari et al.,^2010^]. Thus, even if the exact content of proteins in the immunoprecipitated complexes shown in Figure 1A does not reflect the real *in vivo* situation, as these complexes can be made or broken down during immunoprecipitation, the gel filtration experiment clearly shows that XRCC1 is found in complexes with sizes from 150 to 1500 kDa.

### XRCC1-EYFP Associated Complexes Have LP BER Activity

Immunoprecipitated XRCC1 complexes from S-phase cells have been shown to perform rapid SP BER of uracil and AP sites [Akbari et al.,^2010^]. The gel filtration experiment suggested that XRCC1-associated complexes contain both POLβ and POLδ, a polymerase typically associated with LP BER (Fig. 1A). We therefore examined whether immunoprecipitated XRCC1 complexes from freely cycling cells could perform LP BER using synthetically defined, circular plasmid DNA substrates. We used *N*-ethylmaleimide (NEM) to examine the respective roles of POLβ and POLδ in the DNA repair synthesis activity of XRCC1-EYFP immunoprecipitates. At the concentration used here, adding NEM to the repair reaction inhibits POLδ and several other enzymes but not POLβ [Krokan et al.,^1979^]. Adding NEM inhibited first nucleotide insertion activity by 27% after 15 min of repair, while the degree of inhibition was 57% after 45 min (quantified from the gel in Fig. 1C, left panel). Incorporation of the second nucleotide increased with time in the absence of NEM (up to 20% of total incorporation) but could not be detected in the presence of NEM (Fig. 1C, right panel), suggesting that incorporation of the second nucleotide was performed exclusively by POLδ. Also, neutralising antibodies against POLβ strongly inhibited the total BER capacity (nucleotide insertion and ligation) of the XRCC1-EYFP immunoprecipitates after 15 min of repair (Supporting Information Figure S1C). Together, these results suggest that in the XRCC1-EYFP complexes, POLβ is more important for the initial single nucleotide BER reaction, while POLδ is more important after prolonged incubation and for LP repair synthesis.

To further explore the LP BER activity of XRCC1 complexes, we used a 3-hydroxy-2-hydroxymethyltetrahydrofuran (THF) synthetic AP site substrate. The lyase activity of POLβ is unable to remove 5′-THF residues after APE-1 incision because this lesion is resistant to β-elimination. Thus, repair of THF residues requires LP BER and 5′-flap-endonuclease activity [Kim et al.,^1998^]. The presence of LP BER activity in the XRCC1-EYFP complexes was further analyzed by comparing the repair of DNA substrates containing a normal AP site with substrates containing THF. We analyzed the incorporation of one or two nucleotides and the ability to complete repair (see figure legend and Fig. S1C). Incubating the immunoprecipitated XRCC1-EYFP complexes with the THF substrate in the presence of [α-^33^P] TTP (detects first nucleotide) revealed that a fraction of the repair patch is repaired, including ligation, although in contrast to the normal AP site substrate, a fraction remained unligated in the time frame analyzed (Fig. 1D, compare lanes 1 and 2). This indicates that, although present, LP BER is less efficient than SP BER. As would be expected, a considerable amount of the completely repaired THF substrate consisted of a minimum of two nucleotide insertion products (same substrates in the presence of [α-^33^P] CTP), further supporting the ability of XRCC1-EYFP complexes to perform LP BER (Fig. 1D, lane 4).

### XRCC1, POLβ, and PNK Colocalize with PCNA in Replication Foci

Next, we wanted to study the localisation of the different XRCC1-complexes *in vivo* using live, freely cycling cells expressing XRCC1-EYFP. Recently, low amounts of POLβ and PNK were found in XRCC1-EYFP complexes immunoprecipitated from S-phase cells, suggesting their presence in replication associated XRCC1 complexes [Akbari et al.,^2010^]. POLβ has been reported to interact with PCNA *in vitro* [Kedar et al.,^2002^]; however, neither POLβ nor PNK have been shown to colocalize with replication foci in live untreated cells.

**Fig. 2:**
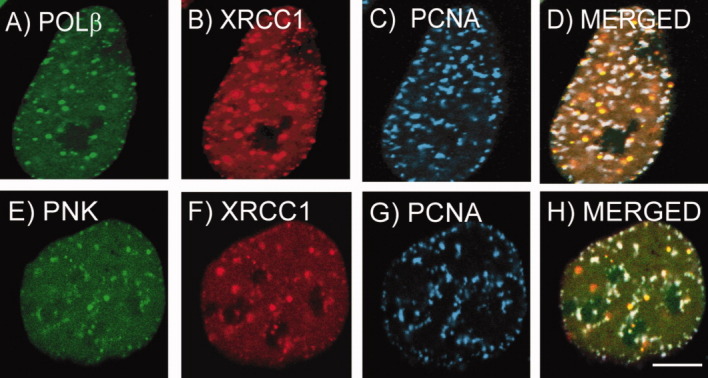
Colocalization of POLβ and PNK with XRCC1 in replication foci. (**A**–**D**) Cotransfection of HeLa cells expressing XRCC1-EYFP with ECFP-POLβ or (**E**–**H**) ECFP-PNK together with HcRed-tagged PCNA as a replication marker. White spots (merged) demonstrate colocalization of POLβ, XRCC1, PCNA (D) and PNK, XRCC1, PCNA (H). Yellow spots show colocalization of POLβ and XRCC1 (D) and PNK and XRCC1 (H). Bars, 5 μm.

Stable XRCC1-EYFP–expressing cells were transfected with POLβ-ECFP or PNK-ECFP and a HcRed-tagged PCNA as an S-phase marker [Leonhardt et al.,^2000^] (Fig. 2). The white spots in S-phase cells indicate colocalization of POLβ or PNK with both XRCC1 and PCNA, whereas yellow spots contain only POLβ or PNK and XRCC1 (Figs. 2D and 2H). Thus, our results identify XRCC1-foci containing PNK and POLβ both outside (yellow) and within replication foci (white) in S-phase cells. We identified a similar colocalization pattern in early, mid- and late S-phase cells judged by the pattern of PCNA foci described previously [Leonhardt et al.,^2000^] (data not shown).

### DNA Damage Induced XRCC1-Foci Contain POLβ and PNK, But Not PCNA

Neither PCNA nor UNG2 is found in XRCC1-foci in undamaged non-S-phase cells [Fan et al.,^2004^; Akbari et al.,^2010^]. XRCC1-foci lacking PCNA possibly represent sites of ongoing SSBR/SP BER of endogenously generated DNA damage, e.g., of the more than the 10^4^ AP sites that are believed to be generated per cell per day, or they may represent constitutively present DNA repair complexes in the absence of DNA damage. However, PCNA was previously reported to colocalize with XRCC1 in micro-irradiation induced foci (UV-A; 365 nm, the exact dose used in this experiment was not specified) [Lan et al.,^2004^]. Thus, whether PCNA is present only at DNA–damage induced foci or whether its presence depends on the type of DNA damage is unclear. In this study, we determined the presence of different BER proteins, including POLβ, PNK, PARP-1, FEN-1, and PCNA, in XRCC1-foci before and after micro-irradiation of two regions with “high” or “low” doses of a 405-nm laser (energy output is given in Materials and Methods).

The role of XRCC1 in the recruitment of DNA repair proteins to sites of DNA damage was first examined using the XRCC1 deficient Chinese hamster ovary cell line (CHO EM9), its parental wild-type cell line (AA8), and CHO EM9 cells expressing human XRCC1-EYFP. We found that localization of POLβ to sites of irradiation was completely dependent on XRCC1, while the recruitment of PNK, PCNA, and PARP-1 were enhanced in the presence of XRCC1 or overexpressed human XRCC1-EYFP (Table I). The increased recruitment of PARP-1 in the presence of overexpressed XRCC1-EYFP, compared with AA8 carrying endogenous levels of XRCC1, may be due to direct interaction between PARP-1 and overexpressed XRCC1.

**Table I:** Micro-Irradiation in Three Different Cell Lines with Different Levels of XRCC1

Proteins	EM9 (Xrcc1^−/−^)	AA8 (Xrcc1^+/+^)	EM9 XE (XRCC1-EYFP)
POLβ	^−^	++	++
PNK	+	++	++
PCNA	(+)	+	+
PARP	+	+	++

++ = Foci after micro-irradiation with low dose; + = Foci after micro-irradiation with high dose; (+) = Weak foci after micro-irradiation with high dose.

**Fig. 3:**
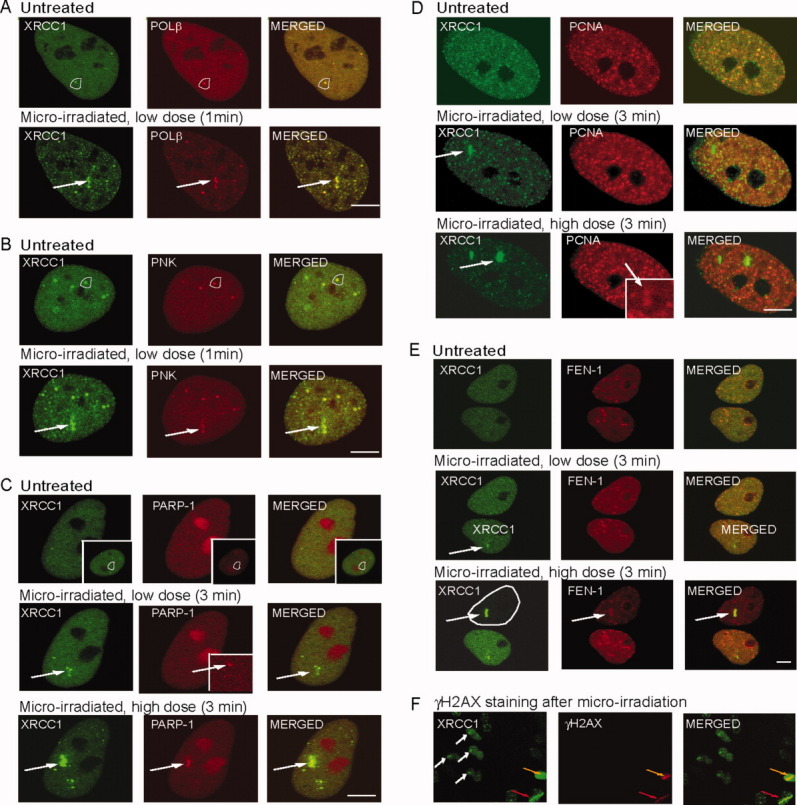
Recruitment of XRCC1, POLβ, PNK, PARP-1, FEN-1, and PCNA to micro-irradiated regions. Cotransfection of HeLa cells with XRCC1-ECFP and EYFP-POLβ (**A**), XRCC1-EYFP and ECFP-PNK (**B**), XRCC1-ECFP and EYFP-PARP-1 (**C**), XRCC1-ECFP and EYFP-PCNA (**D**), XRCC1-ECFP and EYFP-FEN-1 (**E**), XRCC1-EYFP and γH2AX staining (**F**). Micro-irradiation was performed on selected regions of interest with different number of iterations (it) of 1.25(μsec irradiation/pixel (100% output 405 nm laser). White arrows: 50 × 2 pixels, 10 it (“low dose”) or 150 it (“high dose”). Red arrow: 250 × 2 pixels, 600 it. Orange arrow: 150 × 2 pixels, 1500 it. Circles indicate constitutive foci. Bars, 5 μm.

We next performed experiments in HeLa cells. We detected constitutively present XRCC1 foci in undamaged non-S-phase cells that colocalized with POLβ, PNK, and to a more variable degree PARP-1, but not with PCNA (Figs. 3A–3C, and Supporting Information Figure S2A, foci encircled). POLβ, PNK, and PARP-1 are generally considered as SSBR/SP BER proteins, and thus, these foci likely represent complexes carrying out SP repair reactions. When micro-irradiating a selected region with a low dose (10 iterations, see Materials and Methods), we could detect multiple XRCC1-foci in this region within 15 sec (not shown). POLβ and PNK were recruited to these foci in a similar manner as XRCC1, and the foci reached highest intensity after 60–90 sec (Figs. 3A and 3B, the irradiated regions are shown with arrows). Also, multiple additional XRCC1 foci outside the irradiated region (distal XRCC1-foci) emerged (Figs. 3A–3C). These foci were also detected within 15 sec after irradiation, suggesting that they may arise due to rapid intracellular stress signals, possibly mediated by ROS [Bedard and Krause,^2007^; Yalcin et al.,^2010^; Ha et al.,^2011^]. PARP-1 foci could be detected 3 min post-irradiation at low doses (Fig. 3C, mid row, inset). Increasing the dose of micro-irradiation 15-fold (150 iterations) generated stronger XRCC1-foci, and PARP-1 foci could be detected after 60–90 sec (not shown), becoming brighter and easily detectable after 3 min (Fig. 3C, lower row). The same pattern of recruitment was seen when cells were irradiated in the nucleolar region, even though these regions contain higher levels of PARP-1 (data not shown). These results imply that recruitment of XRCC1 is independent of PARP-1; however, because HeLa cells have high levels of endogenous PARP-1, the total amount of PARP-1 needed at the site of DNA damage could also be saturated by endogenous untagged PARP-1.

Next, we examined the recruitment of the LP BER proteins, PCNA and FEN-1, to XRCC1-foci induced by micro-irradiation. Detection of PCNA and FEN-1 at the site of micro-irradiation required 10- to 15-fold higher doses (defined here as our “high dose”) than needed for detection of the SSBR/SP BER proteins POLβ, PNK, and PARP-1 (Figs. 3D and 3E). Additionally, PCNA is not normally found in XRCC1-foci in undamaged non-S-phase cells (Supporting Information Figure S2A and [Fan et al.,^2004^; Akbari et al.,^2010^], suggesting that LP BER proteins are recruited mainly after more extensive DNA damage. These results show that application of an appropriate dose of micro-irradiation is crucial and should be carefully determined in these types of studies; otherwise there is a risk of drawing faulty conclusions related to relocation and involvement of different repair proteins in repair of different DNA damage.

Since irradiation of cells with UV-A beams (320–400 nm) generates reactive oxygen species (ROS), pyrimidine dimers, and double strand breaks (DSBs), depending on the intensity and dose of irradiation [Herrlich et al.,^2008^], we further explored whether the doses of micro-irradiation used herein induced the formation of DSBs by staining irradiated HeLa cells with antibodies against γH2AX (a marker of DSBs). We found that irradiation of HeLa cells with our “high dose” did not induce γH2AX staining, while irradiating larger regions with doses exceeding our “high dose” in the same dish gave positive staining (Fig. 3F, white arrows show foci after “high dose” irradiation, red and orange arrows show foci after irradiation with 4- and 10-fold higher laser doses, respectively). Since a higher dose (5×) was required to induce XRCC1 foci in CHO cells compared to HeLa cells, the dose found to promote the recruitment of PCNA in CHO (i.e., 600 laser beam iterations, Table I) might have actually introduced DSBs.

Analogous to the recruitment of BER proteins to low-dose micro-irradiated regions, XRCC1-foci induced by low doses of MMS or H_2_O_2_ contained POLβ and PNK, but not PCNA (Supporting Information Figure S2), suggesting that the type of DNA damage induced by these three treatment paradigms is comparable. However, the type of DNA damage is not identical in the three cases, as illustrated by the observation that H_2_O_2_ and MMS-induced XRCC1-foci displayed different kinetics (Supporting Information Figure S3B).

### Inhibition of PARylation Causes Only Minor Changes to the Recruitment of SP BER Proteins to Damaged Regions after Low Dose Micro-Irradiation

Our results (Table I and Fig. 3C) indicate that the level of XRCC1 in the foci affects the recruitment and/or the retention of PARP-1 in micro-irradiated regions. To explore whether this was due to the level of polyADP-ribosylation (PARylation), we examined the effect of PARP-inhibitors on the recruitment of DNA repair proteins to the site of DNA damage. We carried out the same experiments as in Figure 3, but in the presence of a PARP-inhibitors (either 4-amino-1,8-naphthalimide (4-AN) or *N*-(5,6-dihydro-6-oxo-2-phenanthridinyl)−2-acetamide hydrochloride (PJ34)). 4-AN and PJ34 have been used in several studies examining the role of PARP and PARylation in BER as well as in *in vivo* models [Abdelkarim et al.,^2001^; Godon et al.,^2008^; Heacock et al.,^2010^; Jelezcova et al.,^2010^]. In the experiments, we used 10 μ*M* of either PARP-inhibitor. This was a concentration that was sufficient and necessary for total abolishment of nuclear PARylation in MMS-treated HeLa cells (Supporting Information Figure S4). Inhibition of PARylation is believed to inhibit dissociation of PARP-1 from DNA, as it has been shown that treatment of 4-AN leads to increased accumulation of PARP-1 and PCNA at sites of micro-irradiation [Godon et al.,^2008^].

**Fig. 4:**
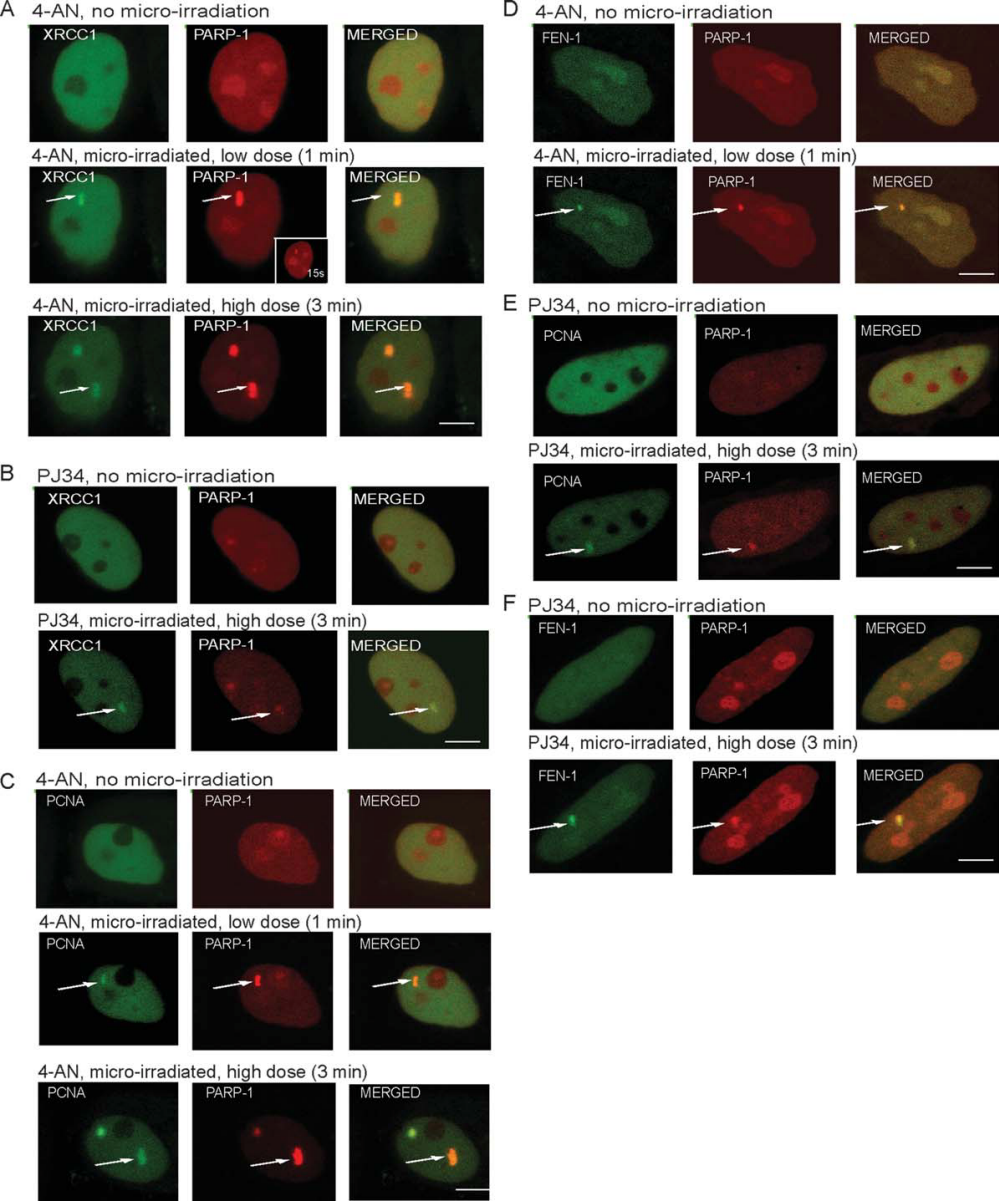
Recruitment of XRCC1, PARP-1, FEN-1, and PCNA to micro-irradiated regions in absence and presence of 4-AN and PJ34. Cotransfection of HeLa cells with XRCC1-ECFP and EYFP- PARP-1 in the presence of 4-AN (**A**), XRCC1-ECFP and EYFP-PARP-1 in the presence of PJ34 (**B**), ECFP-PCNA and EYFP-PARP-1 in the presence of 4-AN (**C**), ECFP-FEN-1 and EYFP-PARP-1 in the presence of 4-AN (**D**), ECFP-PCNA and EYFP-PARP-1 in the presence of PJ34 (**E**), ECFP-FEN-1 and EYFP-PARP-1 in the presence of PJ34 (**F**). Micro-irradiation was performed on selected regions of interest (ROI) with size 50 × 2 pixels. These are marked with white arrows; 10 (low dose) or 150 (high dose) iterations of 1.25 (μsec irradiation/pixel (100% output 405 nm laser) were used. Arrows indicate irradiated regions. Bars, 5 μm.

We found that in the presence of 4-AN, PARP-1 foci were detected immediately after low dose micro-irradiation (15 sec, Fig. 4A, mid row, inset). This observation suggests an increase in PARP-1 accumulation compared with what can be observed in the absence of 4-AN (compare with Fig. 3C). However, this outcome was not the case when treating cells with PJ34, although PJ34 also inhibits PARylation. To detect EYFP-PARP-1 at sites of micro-irradiation in PJ34 treated cells, a 15-fold higher dose of laser irradiation was required (Fig. 4B). Thus, inhibiting PARylation is not likely the underlying mechanism for the accumulation of EYFP-PARP-1 at sites of DNA damage, and instead, it may involve a 4-AN mediated change in the affinity of PARP-1 for DNA.

Interestingly, we found that inhibition of PARylation by 4-AN or PJ34 only caused a minor reduction in the recruitment of XRCC1 (Figs. 4A and 4B, left panel). The recruitment of XRCC1 is slightly reduced at the high irradiation dose in the presence of 4-AN, but not reduced at low dose irradiation. Recruitment of XRCC1 is not seen at low dose irradiation and is slightly reduced at high dose in the presence of PJ34 (compare Fig. 3C with Figs. 4A and 4B).

An increased recruitment of both PCNA and FEN-1 was detected in the presence of 4-AN (compare Figs. 3D and 3E with Figs. 4C and 4D), while no (low dose) or very little (high dose) effect of 4-AN treatment was seen for recruitment of XRCC1, POLβ, or PNK (data shown only for XRCC1). Thus, the small change observed at high dose affected all three proteins similarly, suggesting that these repair proteins are regulated similarly and are found in a same sub-complex. PCNA and FEN-1 recruitment was not significantly changed by PJ34 treatment of the cells (compare Figs. 3D and 3E with Figs. 4E and 4F); thus the increase in PCNA and FEN-1 recruitment by 4-AN treatment is likely connected to an increased accumulation of PARP-1 at the site of DNA damage. Our results clearly show that XRCC1/PNK/POLβ are recruited to sites of DNA damage independent of PARP-1 accumulation and PARylation, and interestingly, that different PARP-inhibitors affect DNA repair protein recruitment differently.

## DISCUSSION

BER corrects a variety of DNA base lesions and SSBs that are constantly generated by endogenous and environmental agents. The fact that BER can be reconstituted *in vitro* by a few “core proteins” has contributed to a rather simplified notion of BER. However, in recent years, new findings have given rise to a more complex view of this repair pathway [Hegde et al.,^2008^]. For instance, PCNA and XRCC1 interact with a number of DNA repair proteins and have been suggested to provide a platform for the organization of DNA repair [Mortusewicz and Leonhardt,^2007^]. Our aim for this study was to further elucidate the role of XRCC1 in orchestrating cellular BER/SSBR.

Western blotting of XRCC1-EYFP immunoprecipitates from gel filtration fractions identified the presence of XRCC1 in complexes of sizes between 70 and 2000 kDa. Immunoprecipitates from fractions containing protein complexes with molecular weights between 350 and 700 kDa exhibited the highest repair capacity. Furthermore, we showed that immunoprecipitated XRCC1 complexes from unfractionated nuclear extracts possessed both SP and LP BER capacity. The theoretical molecular weight of an XRCC1 repair complex containing all proteins necessary to carry out both complete SP and LP BER, i.e., Lig III (∼103 kDa), Lig I (∼125 kDa), XRCC1 (70 kDa), APE-1 (35 kDa), FEN-1 (48 kDa), PCNA (32 kDa), and POLβ (39 kDa) or POLδ (125 kDa), is between 350 and 600 kDa. Our gel filtration results are different from a previous report where fractionation of cell extracts only detected XRCC1 in small complexes [Parsons et al.,2005a]. Although we are currently unable to explain these discrepancies, the differences in the methods used for the preparation of cell extracts (e.g., we used DNase/RNase treated nuclear extracts), as well as the conditions of gel filtration, are among the plausible causes.

In a number of studies designed to elucidate the organization of BER and the recruitment of BER proteins to sites of DNA damage, either the exact dose of irradiation was not specified or very high doses of the DNA damaging agent (like H_2_O_2_ or micro-irradiation) were used relative to the doses we have used in this study [Lan et al.,^2004^; Mortusewicz et al.,^2006^, ^2007^; Godon et al.,^2008^]. This may have two important consequences. First, a direct comparison of results from different studies is hampered by the shortage of information about the experimental conditions. Second, as we have shown herein, the dose of the DNA-damaging agent has a pivotal effect on the observed recruitment of repair proteins. Similar concerns have been raised for induction of DSBs by irradiation [Bekker-Jensen et al.,^2006^; Kong et al.,^2009^].

Our data herein indicate that XRCC1 is present in at least three categories of foci, likely involving several different sub-types of XRCC1 complexes: (i) XRCC1 complexes colocalizing with PCNA in replication foci; (ii) XRCC1-complexes that are rapidly formed after treatment of cells with low dose micro-irradiation, H_2_O_2_, or MMS and that are present in untreated cells independent of the cell cycle likely repairing spontaneous DNA damage; and (iii) XRCC1-complexes that are formed after treatment with high dose micro-irradiation or other DNA damaging agents causing high levels of DNA strand breaks. The type of DNA damage and/or cell signalling induced by the dose of micro-irradiation, MMS, or H_2_O_2_, as well as the cell cycle stage, appears to dictate which XRCC1 complex partner(s) are recruited. For example, recruitment of LP BER proteins such as FEN-1 and PCNA required 10–15-fold higher doses of micro-irradiation and which presumably leads to higher levels of ROS and SSBs.

**Fig. 5:**
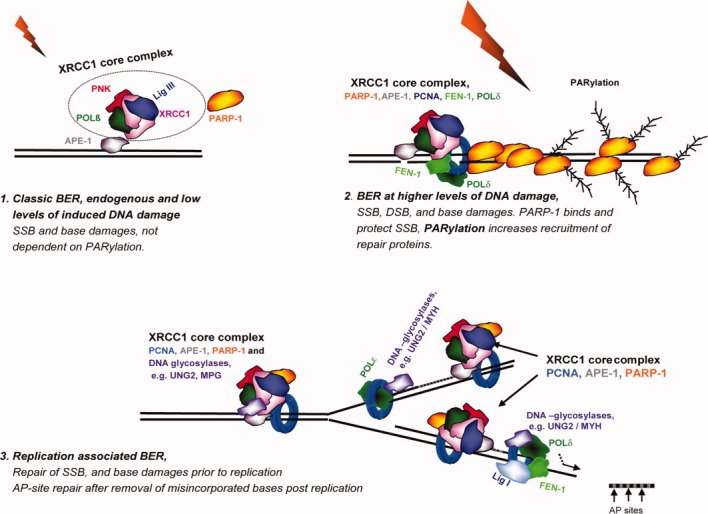
A model of three modes of BER. For simplicity, only a limited number of BER proteins are included in the model.

Studies have begun to reveal that there may exist three modes of BER/SSBR (summarized in Fig. 5). For simplicity only a limited number of proteins and only the BER proteins we have focused on in this and previous work [Akbari et al.,^2010^] are included in the model. We classify the first mode as “classic BER”, involving coordinated hand-off of the substrate/product from the initiating DNA glycosylase to APE-1 to POLβ/PNK/XRCC1/Lig III multi-protein complexes (*M*_w_ ≥ 350 kDa). Classic BER would presumably handle any general genome damage at levels that fall within the repair capacity of the cell, e.g., endogenous AP sites, base damages, and SSBs, as well as most DNA damage introduced by low-dose micro-irradiation, H_2_O_2_, and MMS. Interestingly, we found that the XRCC1 complexes immunoprecipitated from fractions containing protein complexes between 350 and 700 kDa have the highest repair capacity (Fig. 1). Because we detected PARP-1 in a fraction of constitutive XRCC1-foci in the absence of exogenous damage, as well as low levels of PARP-1 in XRCC1-foci after low-dose micro-irradiation (Fig. 3C) and in XRCC1-complexes isolated from S-phase [Akbari et al.,^2010^], PARP-1 and other proteins may also be part of these complexes. However, the classic repair process likely would not engage PARP-1 and PARylation directly, unless it is interrupted and repair intermediates are exposed; thus this repair mode will not be directly affected by inhibition of PARylation.

The second BER/SSBR mode (Fig. 5) would be more specific for higher levels of DSBs, direct or indirect, that exceed cellular repair capacity for rapid repair and may therefore bind PARP-1 as a protective mechanism, as suggested previously [Parsons et al.,2005b; Strom et al.,^2010^]. In this case, subsequent PARylation facilitates the recruitment of repair proteins to the damaged site via interactions through their different PAR-interacting domains, leading to activation of intracellular signalling and inhibition of transcription, etc. Staining for γH2AX were negative after micro-irradiating with our “high dose,” thus we were not introducing DSBs but likely DNA damage repaired by BER/SSBR complexes. However, we detected an increased recruitment of PARP-1, FEN-1, and PCNA using “high dose” irradiation, which may suggest introduction of DNA damages other than those created with “low dose.” The dependence upon PARylation will likely increase with increasing levels of strand breaks; however, because PARP-1-deficient cells do repair SSBs efficiently [Vodenicharov et al.,^2000^] and 90% of the cell's PARylation activity is due to PARP-1 [Megnin-Chanet et al.,^2010^], it is not essential for repair.

The third mode of BER (Fig. 5) would involve larger protein complexes (>700 kDa) and is associated with the DNA replication machinery as described in our previous study [Akbari et al.,^2010^], where we identified two replication associated BER complexes with different protein content: (1) UNG2 associated complexes containing more POLδ and Lig I and (2) XRCC1 associated complexes containing more Lig III, POLβ, PARP-1, and PNK. These complexes had significant differences in their repair activities. Here, we verify the presence of POLβ and PNK in these XRCC1-complexes *in vivo*. A close association of BER with DNA replication is likely important for genome maintenance, and as such, XRCC1 may have an important role in facilitating BER in the vicinity of the replication fork, probably at both pre- and post-replication complexes. As we did not observe localisation of UNG2 in XRCC1-foci of non-S-phase cells [Akbari et al.,^2010^], and because UNG2, MPG, or hOGG1 are not recruited to XRCC1 foci after micro-irradiation (data not shown), we conclude that these DNA glycosylases are not always a stable partner of XRCC1 complexes even though they do interact with XRCC1 [Marsin et al.,^2003^; Campalans et al.,^2005^; Akbari et al.,^2010^].

Surprisingly, we found that the two PARP-inhibitors, 4-AN and PJ34, affected PARP-1 recruitment and/or accumulation at sites of DNA damage differently after micro-irradiation. Although the molecular basis for this difference is unclear, we did find that PARylation was dispensable for subsequent protein recruitment, and that the amount of PARP-1 present at the site of DNA damage affected the repertoire of repair proteins recruited, as shown for PCNA and FEN-1. An increased sensitivity toward MMS is seen when treating cells with PJ34 [Jelezcova et al.,^2010^], and PJ34 exposure does not lead to accumulation of PARP-1 at sites of DNA damage (Figs. 4B–4E); thus, the hypothesis that PARP inhibition reduces BER because of an accumulation of PARP-1 at the site of DNA damage due to lack of PARylation [Godon et al.,^2008^] has to be reconsidered. Recently, the function of PARP-1 in BER/SSBR [Jelezcova et al.,^2010^; Strom et al.,^2010^], in signal transduction [Stilmann et al.,^2009^], chromatin remodelling, and in regulating transcription [Godon et al.,^2008^; Lonn et al.,^2010^] has been reconsidered. The overall message is that several important cellular regulatory mechanisms are likely to be directed by PARylation, and consequently also influenced by PARP-inhibition. This fact makes it difficult to interpret the actual function of the PARylation in SSBR/BER. As targeting PARylation in cancer therapy is showing great promise, knowledge about the cellular consequences of inhibition of PARylation is becoming more important. Thus, our results have implications for future design of DNA repair studies as well as for our understanding of the molecular mechanisms of PARP-inhibitors.
