# Supplementary material for: XRCC1 Coordinates Disparate Responses and Multiprotein Repair Complexes Depending on the Nature and Context of the DNA Damage
Source: Environ Mol Mutagen. 2011 Jul 22;52(8):623–35. doi: 10.1002/em.20663 (PMC3229989; doi:10.1002/em.20663)
Supplement: Supplementary file 1 [file em0052-0623-SD1.doc]

**Supplemental Figure S1**

**
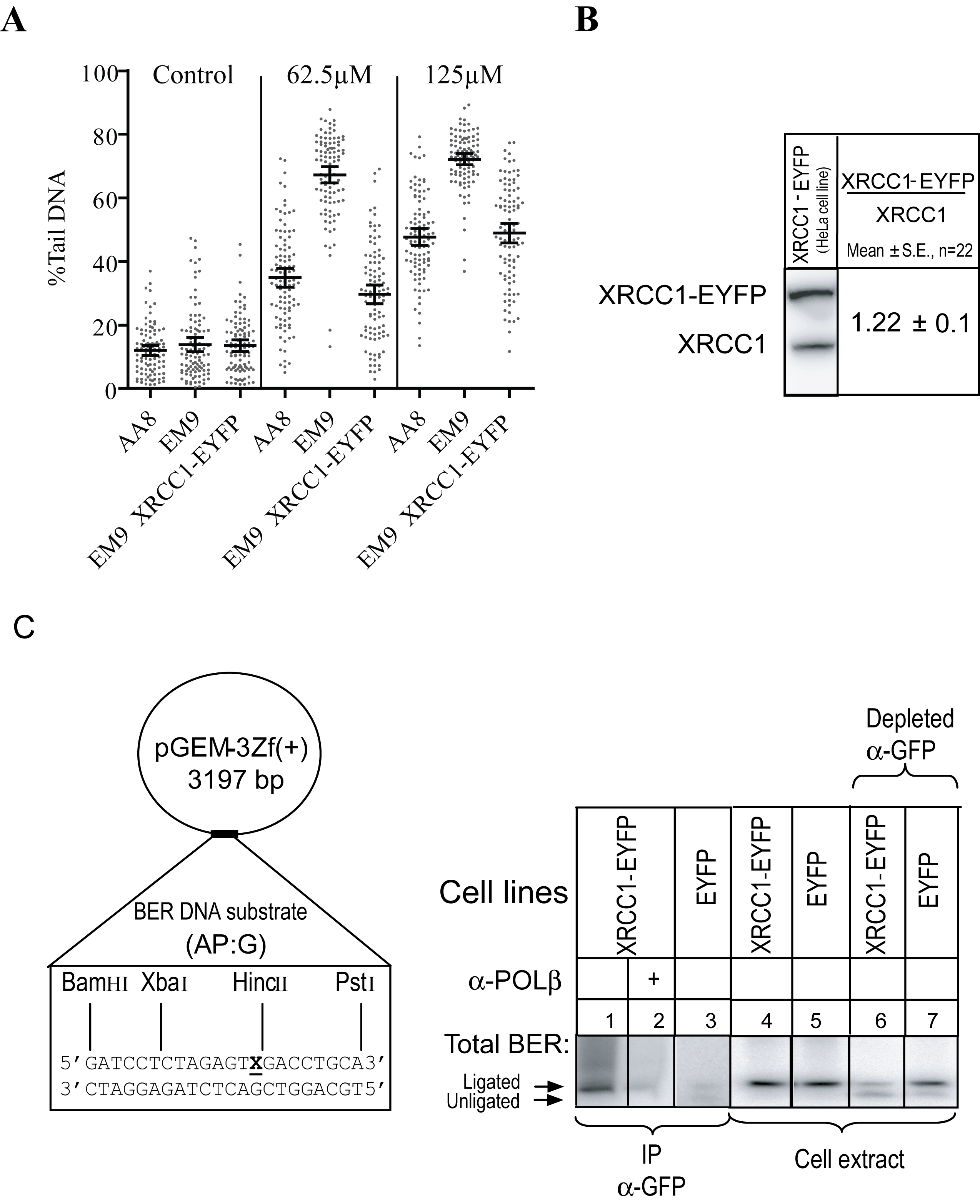
**

**Figure S1: Single-cell alkaline gel electrophoresis (Comet assay)** (A) analysis of CHO AA8 (wild-type), CHO EM9 (Xrcc1^-^/^-^*)* and CHO EM9 XRCC1-EYFP cells after treatment with different doses of H_2_O_2_ for 10 min and zero time recovery. The cells were then washed twice with PBS and harvested. The control cells were mock treated. The cells were harvested by centrifugation at 400 × *g* for 5 min, embedded in 1% low melting point agarose and lysed overnight at 4 °C in lysis solution (2.5 M NaCl, 0.1 M EDTA, 10 mM Tris, 10% DMSO, 1% Triton X-100, 17 mM Na Lauroyl Sarcosine, pH 10). Alkaline (pH 13.3) single-cell gel electrophoresis (Comet assay) was performed as described [1], except the samples were not treated with uracil DNA-glycosylase. 100 Comets were selected randomly from each slide and evaluated using Komet 5.0 Imaging Software (Andor Technology). Data presented as scatter plot of % Tail DNA on the y-axis. Mean with 95% Confidence Interval error bars are given. **Content and functionality of XRCC1-EYFP complexes.** (B) Western analysis showing the expression levels of XRCC1-EYFP (C) Left panel: schematic illustration of DNA substrate for BER analysis containing AP site (X) (see Fig. S1C). Right panel: lane 1; BER of immunoprecipitated XRCC1-EYFP, lane 2; BER of immunoprecipitated XRCC1-EYFP in the presence of neutralising α-POLβ antibodies, lane 3; BER of immunoprecipitates from EYFP-expressing cell extract as control. BER of cell extracts before (lanes 4 and 5) and after immuno-depletion (lanes 6 and 7) using α-GFP. Extract from EYFP-expressing cells was used as control. Complete repair of the repair patch are analysed digestion with BamHI and PstI, cutting 8 nucleotides downstream the site of incorporation (X) in a cccDNA substrate.

XRCC1 interacts with many SSBR and BER proteins but the exact role of XRCC1 as a scaffold protein in BER has remained elusive. We established a HeLa cell line stably expressing near endogenous levels of EYFP tagged, functional active XRCC1 as measured by complementation experiments using the XRCC1 deficient of CHO EM9 cell line, (XRCC1-EYFP) (Supplemental Fig. S1A and B). We used extracts from these cells to immunoprecipitate XRCC1 associated proteins by a tag-specific antibody. Although XRCC1 has been shown to interact with DNA repair proteins, the presence of XRCC1 complexes capable of conducting complete BER has not been reported. To assess the functional capacity of the immunoprecipitated complexes, we examined the ability of XRCC1-EYFP immunoprecipitates to carry out repair of AP sites. Fig. S1C, left panel, illustrates the substrate used for BER analysis. The XRCC1-EYFP immunoprecipitates displayed the ability for complete repair of AP site, while immunoprecipitates prepared from the EYFP-expressing cell extract did not (Fig. S1C, lane 1 and 3, respectively). The AP site repair capacity of the immunoprecipitates from the XRCC1-EYFP extracts was significantly inhibited by neutralising POLβ-antibodies (lane 2), supporting that POLβ is an important repair polymerase in the XRCC1-EYFP complexes. This experiment also shows that other “core BER proteins”, e.g. enzymes with AP-endonuclease and ligase activity, are present in the XRCC1 multiprotein BER complexes. This conclusion was further supported by analysis of the XRCC1-EYFP depleted extract, which showed a considerable reduction in BER activity (Fig. S1C, lane 6).

**Supplemental Figure S2**


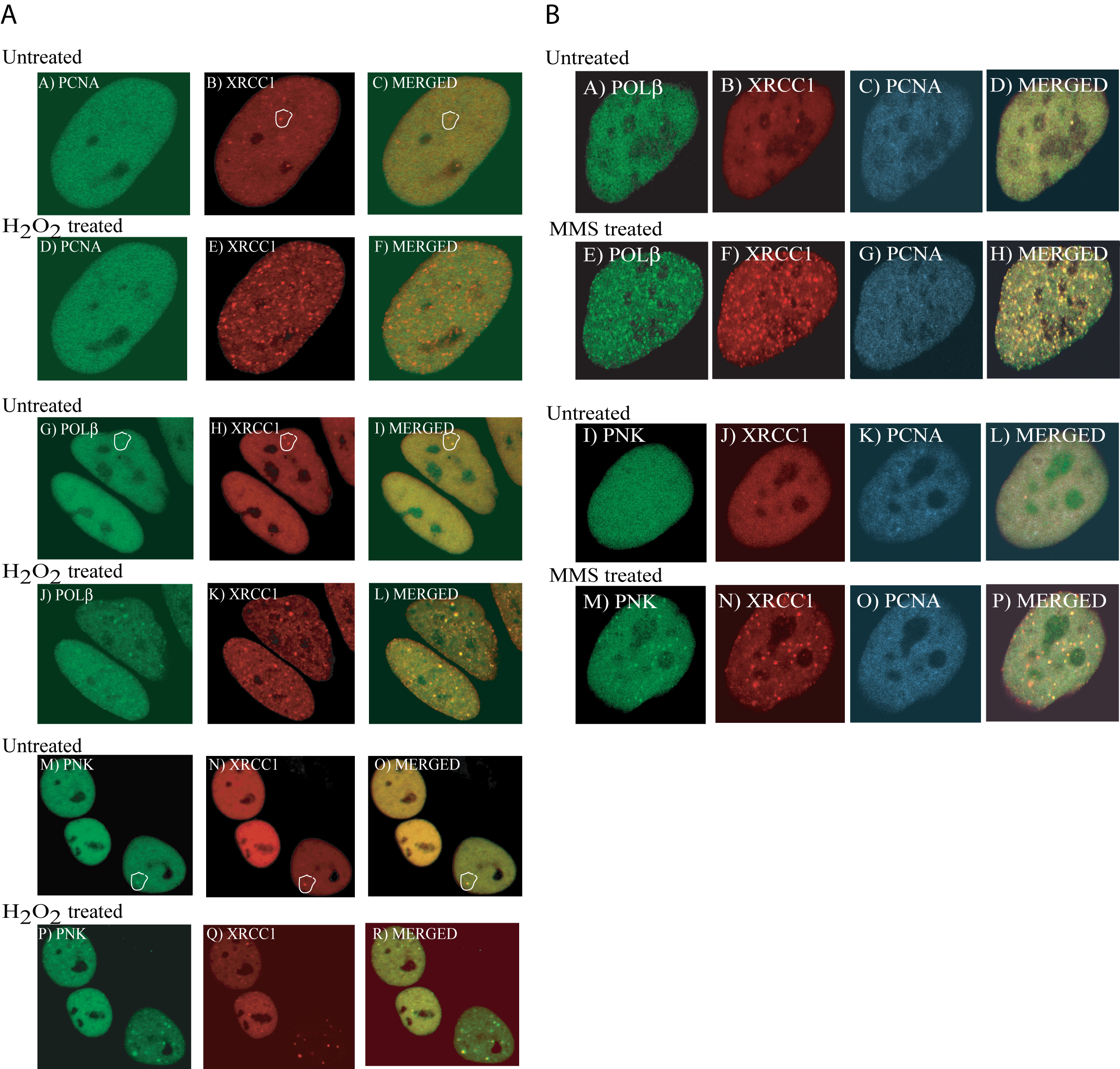


**Figure S2:** (A) **Co-localisation of POLβ and PNK, but not PCNA, in H_2_O_2_ induced XRCC1-EYFP foci.** Co-transfection of HeLa cells with XRCC1-EYFP and ECFP-PCNA (1-6), XRCC1-EYFP and ECFP-POLβ (7-12), and XRCC1-EYFP and ECFP-PNK (13-18). The cells were treated with H_2_O_2_ (62.5 μM) and examined for co-localisation after 10 min (4-6, 10-12 and 16-18). Co-localisation of ECFP-POLβ and ECFP-PNK with XRCC1-EYFP (12 and 18, respectively) are seen as yellow spots. (B) **Co-localisation of POLβ and PNK, but not PCNA, in MMS induced non-S-phase XRCC1-EYFP foci.** Co-transfection of HeLa cells stably expressing XRCC1-EYFP with ECFP-POLβ (1-8) or ECFP-PNK (9-16) both with HcRed-tagged PCNA as a replication marker. (5-8, 13-16) Foci formation after MMS treatment (600 μM) for 30 min. Co-localisation of ECFP-POLβ and XRCC1-EYFP (8) and ECFP-PNK and XRCC1-EYFP (16), are seen as yellow spots.

Analogous to DNA damage induced XRCC1 complexes made by low dose micro-irradiation, we examined whether XRCC1-complexes induced by low doses of MMS and H_2_O_2_ contained PCNA. Next, we co-transfected HeLa cells with XRCC1-EYFP and ECFP-PCNA and analysed non-S-phase cells, i.e. cells without detectable PCNA foci (Figure S2A). In these cells, no co-localisation of ECFP-PCNA with XRCC1-EYFP-foci could be observed 10 min after H_2_O_2_ treatment (Figure S2A, picture 4 to 6). Notably, under similar conditions, both POLβ and PNK were detected in the H_2_O_2_ induced XRCC1-EYFP-foci (Figure S2A, picture 10-12 and 16-18, respectively). Also, XRCC1-foci in non-treated apparently non-S-phase cells contained POLß and PNK (Figure S2A, picture 7-9 and 13-15, white arrows). We observed a similar pattern of response upon MMS treatment, i.e. the number of foci containing co-localised XRCC1-POLβ (Figure S2B, picture 5-8) and XRCC1-PNK (Figure S2B, picture 13-16) increased 30 min after exposure to MMS, while no PCNA foci were detected (Figure S2B, picture 7 and 15).

Our results suggest the presence of three distinct types of XRCC1 foci; 1) constitutively present foci containing POLβ and PNK; 2) foci induced by DNA damage, containing POLβ and PNK, and; 3) replication associated foci containing POLβ, PNK and PCNA. Neither 1) or 2) contains PCNA,

**Supplemental Figure S3:**

*
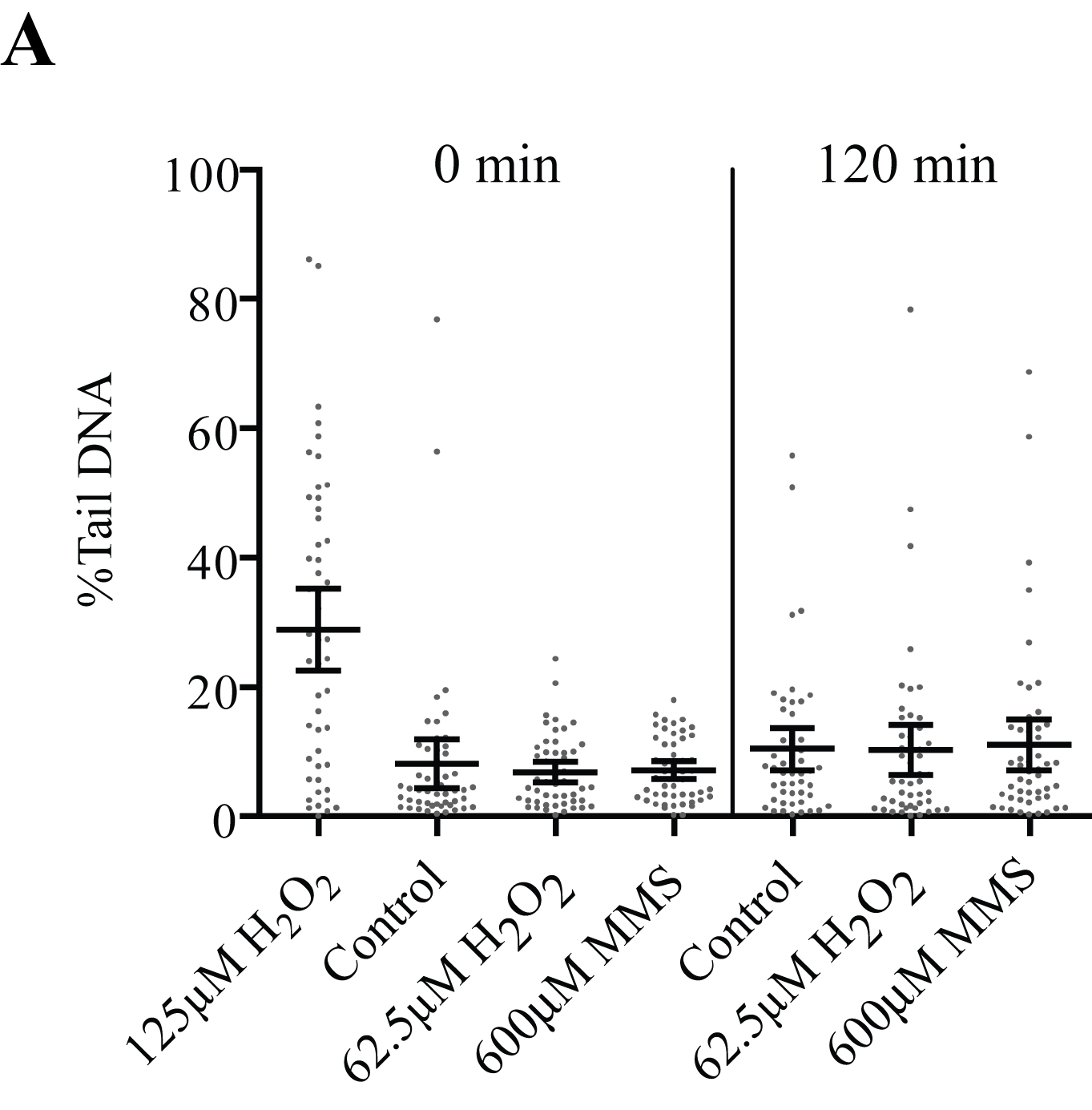
*

**B**

**Panel:**

**I II III IV**


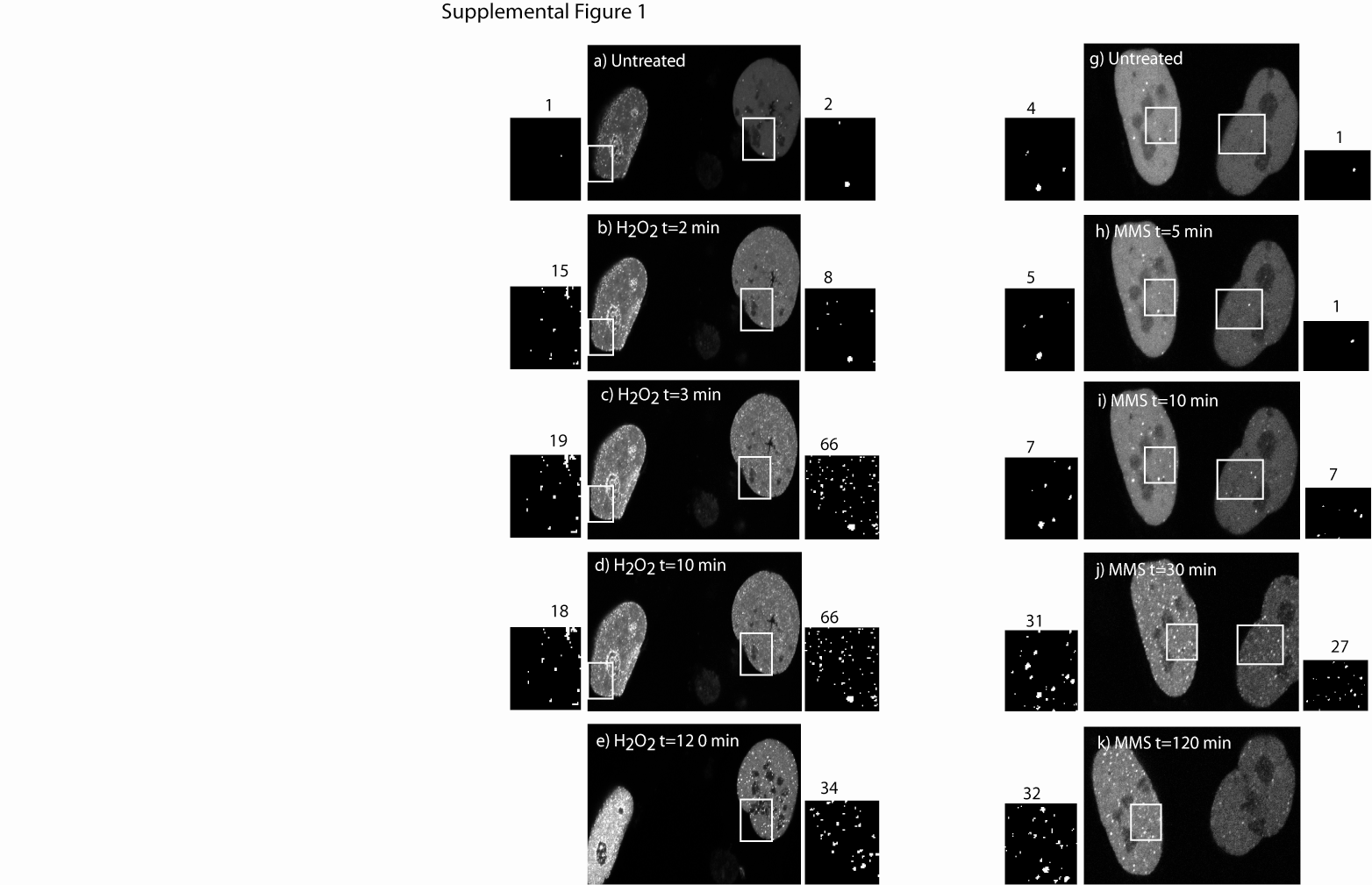


**Figure S3.** **Characterisation of XRCC1-EYFP fusion protein and the stably expressing XRCC1-EYFP cell line.** (A) HeLa cells expressing XRCC1-EYFP were treated with 125 μM H_2_O_2_ (positive control), 62.5 μM H_2_O_2_ or 600 μM MMS for 10 min. Untreated cells were included as a negative control. The post-treatment DNA repair was monitored using the Comet assay at the indicated times. Data presented as scatter plot of % Tail DNA on the y-axis. Mean with 95% Confidence Interval error bars are given. (B) **Time-response study of XRCC1 foci formation in freely cycling living cells after H_2_O_2_ and MMS treatment.** (a and g) untreated HeLa cells stably expressing XRCC1-EYFP fusion protein; (b-e) the same cells treated with H_2_O_2_ (62.5 μM) and (h-k) with MMS (600 μM), respectively. The formation of foci was studied at the indicated times post-treatment. Each figure shows two cells. Regions within the cells, marked with white boxes in a-k, are enlarged and presented as pictures (panel I, II III and IV) where pixels above a certain threshold levels are shown in white. The foci defined by white pixels, one single or several connected pixels, are counted as one unit. Thresholds are set according to intensity of the fluorescence, and are 200 in panel I, 128 in panel II, 178 in panel III and 110 in panel IV.

No area is selected in the right cell in e) and in the right cell in k) due to movement of these cell out of the focal plan.

Exposure of XRCC1-EYFP expressing cells to 62.5 μM of H_2_O_2_ or 600 μM MMS for 10 min did not induce DNA damage detectable by the Comet assay, while H_2_O_2_ at 125 μM induced significant amount of DNA damage (Figure S3A, 0 min recovery). However, we found that 62.5 μM H_2_O_2_ and 600 μM MMS were both sufficient to induce formation of XRCC1-EYFP foci within 10 min. Notably, the kinetics of foci formation was slightly different between H_2_O_2_ and MMS; H_2_O_2_ induced foci reached a maximum number after 2-3 min, while the number of foci induced by MMS peaked after 30 min (shown in Figure S2B). This likely reflects different DNA damages induced by the different agents. Figure S2a and g, (S2a, S2g), shows images of two cells in a freely cycling cell population prior to addition of H_2_O_2_ and MMS. After addition of H_2_O_2_, the number of small XRCC1-EYFP foci in the cells increased significantly within two min (S2b). The number of foci within the cells treated peaked between 2-3 min after the addition of H_2_O_2_, and remained stable for at least 10 min (S2d). After 60 min they gradually declined in number and after 120 min we detected fewer, but on average, larger foci (S2e). Next, we examined formation of XRCC1 foci as a function of time after MMS treatment (S1, h-k). An increased number of foci were detected after 10 min and the number of foci reached a maximum 30 min after MMS exposure. This was a considerably slower kinetics than seen for the H_2_O_2_ induced foci, which peaked between 2-3 min (compare c with j). Unlike H_2_O_2_ treatment, a similar number of foci were visible 120 min and 210 min after MMS exposure (S1k and data not shown), indicating a slower repair of these damages than those induced by H_2_O_2_.

All XRCC1-EYFP expressing cells in dishes treated with H_2_O_2_ or MMS contained numerous foci after one scan in the confocal microscope, while less than 30% (the majority most likely S-phase cells) of the cells in parallel non-treated dishes displayed a high number of “spontaneous” XRCC1 foci (not shown). Thus, the increased number of foci was not caused by the scanning itself, but was a direct consequence of H_2_O_2_ or MMS treatment.

These results show that the exposure of the cells to low doses of H_2_O_2_ and MMS, which did not generate sufficient levels of DNA damage to be detectable by the Comet assay (Supplemental Figure S3A), induced rapid formation of numerous XRCC1-EYFP foci that likely represent BER complexes.

**Supplemental Figure S4:**


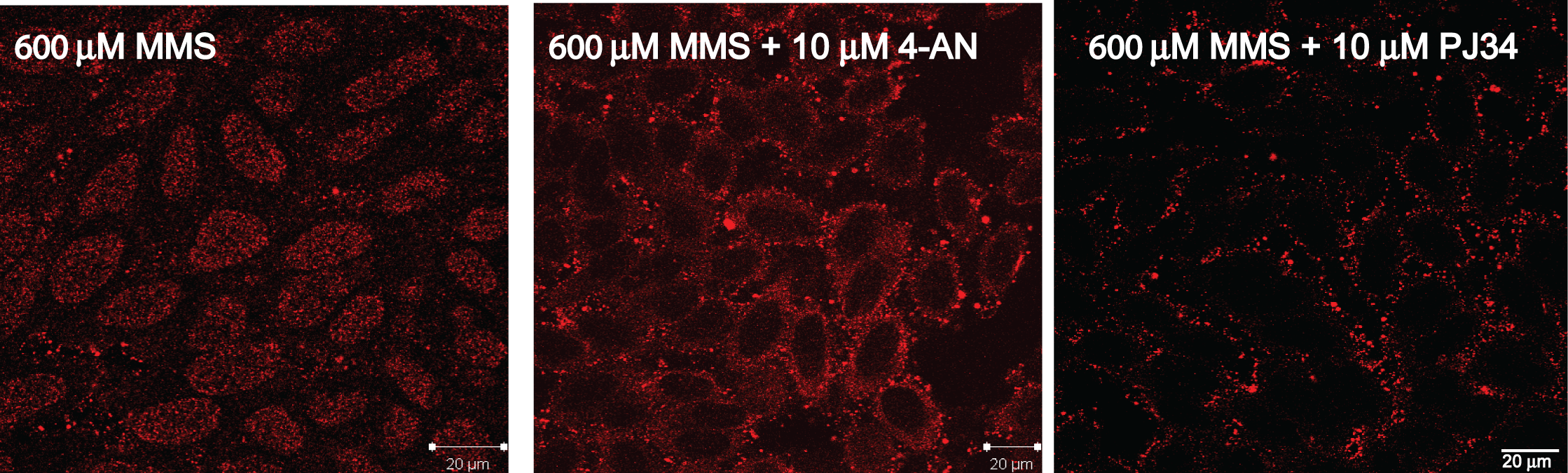


**Figure S4. Inhbition of PARylation by 4-AN and PJ34.** Cells were treated 1 h with either 10% DMSO (mock), 10 μM 4-AN or 10 μM PJ34 prior to a 10 min treatment with 600 μM MMS in phosphate-buffered saline (PBS). Cells were then washed once with PBS, fixed with 2% paraformaldehyde on ice for 15 min, washed once with PBS, permeabilized with methanol at -20 °C for 30 min, washed once with PBS-FCS (2% fetal calf serum in PBS) and blocked by incubation in PBS-FCS for 30 min prior to incubation with antibodies. Primary (mouse monoclonal α-PAR, Trevigen) and secondary (Alexa-Fluor-532 goat anti-mouse, Invitrogen) antibodies were diluted 1:200 and 1:4000 in PBS-FCS respectively. The fixed cells where incubated at room temperature overnight with primary antibodies and 30 min with secondary antibodies.

Supplemental Figure 4 shows that 10 μM 4-AN and PJ34 totally abolished the nuclear PARylation. Under our experimental conditions 1 μM PJ34 did not completely shut down PARylation, and recruitment of XRCC1 to micro-irradiated regions was also seen in presence of 100 μM PJ34.

**References:**

[1] H. Nilsen, I. Rosewell, P. Robins, C.F. Skjelbred, S. Andersen, G. Slupphaug, G. Daly, H.E. Krokan, T. Lindahl and D.E. Barnes Uracil-DNA glycosylase (UNG)-deficient mice reveal a primary role of the enzyme during DNA replication, Mol. Cell 5 (2000) 1059-1065.
